# Comparative cytokine profiling in cerebrospinal fluid and serum of dogs with meningoencephalitis of unknown origin

**DOI:** 10.1093/jvimsj/aalag248

**Published:** 2026-11-10

**Authors:** Aurelia Gasser, Thomas Démoulins, Veronika M Stein

**Affiliations:** Division of Clinical Neurology, Department of Clinical Veterinary Medicine, Vetsuisse Faculty, University of Bern, Bern 3012, Switzerland; Institute of Veterinary Bacteriology, Department of Infectious Diseases and Pathobiology, Vetsuisse Faculty, University of Bern, Bern 3001, Switzerland; Division of Clinical Neurology, Department of Clinical Veterinary Medicine, Vetsuisse Faculty, University of Bern, Bern 3012, Switzerland

**Keywords:** canine, biomarkers, multiplex immunoassay, neuroinflammation, pro-inflammatory mediators

## Abstract

**Background:**

Meningoencephalitis of unknown origin (MUO) is a common inflammatory central nervous system (CNS) disease in dogs with unclear etiology, complicating diagnosis.

**Hypothesis/Objectives:**

Dogs with MUO exhibit distinct cytokine profiles in cerebrospinal fluid (CSF) compared to other CNS diseases and controls.

**Animals:**

The study included 23 dogs diagnosed with MUO based on clinical signs, supportive CSF, and imaging findings. For comparison, 110 dogs were included, comprising dogs with CNS disorders, neurological diseases not involving the CNS (non-CNS), and orthopedic diseases.

**Methods:**

In this prospective cross-sectional observational study, 6 cytokines (interferon-γ [IFN-γ], TNF-α, IL-2, IL-6, IL-10, IL-15) were measured in paired CSF and serum samples using multiplex bead-based immunoassays. Primary outcome was the association between CSF cytokine concentrations and MUO, assessed by logistic regression.

**Results:**

Meningoencephalitis of unknown origin showed significantly higher CSF concentrations compared to idiopathic epilepsy, intracranial neoplasia, intervertebral disc herniation, and non-CNS diseases in IFN-γ (MUO: median 108.4 pg/mL, IQR 25.8 vs comparison group: 10.5 pg/mL, IQR 0.0; β-coefficient 0.039, 95% CI, 0.021-0.057, *P* < .001) and interleukin (IL)-10 (MUO: 171.1 pg/mL, IQR 37.0 vs 8.5 pg/mL, IQR 0.0; β-coefficient 0.021, 95% CI, 0.011-0.030, *P* < .001). Moreover, TNF-α (β-coefficient 0.062, 95% CI, 0.023-0.102, *P* < .01), IL-15 (β-coefficient 0.080, 95% CI, 0.012-0.148, *P* < .05) and IL-2 (β-coefficient 0.055, 95% CI, 0.005-0.104, *P* < .05) concentrations were also elevated in MUO versus comparison group.

**Conclusions and clinical importance:**

Cerebrospinal fluid cytokine profiling identifies a characteristic intrathecal immune signature in MUO, advancing the understanding of its immunopathogenesis.

## Introduction

Meningoencephalitis of unknown origin (MUO) is one of the most prevalent non-infectious inflammatory diseases of the canine central nervous system (CNS). Despite its clinical relevance, the pathogenesis of MUO remains incompletely understood, and no disease-specific diagnostic test is currently available. Diagnosis is typically based on a combination of clinical signs, advanced imaging, cerebrospinal fluid (CSF) analysis, and exclusion of infectious causes.^1^

Cytokines including interleukins (ILs), interferon-γ (IFN-γ), tumor necrosis factor α (TNF-α), and related molecules have been investigated as potential biomarkers and to improve understanding of MUO immunopathogenesis.^2–7^

In MUO subtypes, IFN-γ predominates in necrotizing meningoencephalitis (NME), whereas IL-17 and IL-4 are predominant in granulomatous meningoencephalitis (GME).^7^ Peripheral blood analyses showed reduced IFN-γ and increased CD4-positive IL-17 T-cells.^3^ A recent study detected IFN-γ, TNF-α, IL-2, IL-15, and granulocyte–macrophage colony-stimulating factor exclusively in CSF of MUO dogs, with additionally elevated IL-6, IL-8, IL-18, and monocyte chemoattractant protein-1 compared to idiopathic epilepsy (IE) and intracranial neoplasia.^5^

Meningoencephalitis of unknown origin in dogs shares several pathophysiological features with multiple sclerosis (MS) in humans, characterized by B- and T-cell mediated neuroinflammation and neurodegeneration. Shared features include mononuclear CSF pleocytosis, multifocal T2-hyperintense CNS lesions on magnetic resonance imaging (MRI), CSF-specific oligoclonal bands,^8^ elevated neurofilament light chain concentrations,^9^^,^^10^ abnormal τ hyperphosphorylation in inflammatory lesions,^11^ and reduced abundance of anti-inflammatory gut bacteria.^12^ Meta-analyses in MS have demonstrated consistent elevations of TNF-α, IFN-γ, IL-2, and IL-15 in CSF and blood, while IL-6 and IL-10 show variable patterns, suggesting similar cytokine dysregulation in MUO in dogs.^13–15^

Cytokine analyses in steroid-responsive meningitis-arteritis (SRMA) have consistently demonstrated elevated IL-6, IL-8, IL-17, transforming growth factor β, and vascular endothelial growth factor in CSF,^4^^,^^6^^,^^16–20^ emphasizing the need for similar investigations in MUO to explore disease-specific immunological profiles.

Further characterization of cytokine patterns in CSF and serum may improve understanding of MUO immunopathogenesis and address the need for reliable biomarkers for ante-mortem diagnostics.

We hypothesize that cytokine concentrations in the CSF are elevated in dogs with MUO compared to those with other neurological conditions, such as IE, intracranial neoplasia, SRMA, and intervertebral disc herniation (IVDH), as well as to a control group of neurologic dogs without CNS involvement (non-CNS) and orthopedic disease controls. Moreover, we hypothesize that MUO and SRMA exhibit distinct disease-specific cytokine profiles in CSF and serum, reflecting different immune activation patterns.

## Materials and methods

### Study sample and design

This prospective cross-sectional observational study (2018-2023) included dogs with a clinical diagnosis of MUO (*n* = 23), based on clinical findings, supportive CSF and MRI findings, and exclusion of relevant infectious diseases. In 3 dogs (3/23), the diagnosis of MUO was confirmed histopathologically. Dogs with other neurological disorders, comprising SRMA (*n* = 22), IE (*n* = 22), intracranial neoplasia (*n* = 21), and IVDH (*n* = 19) served for comparison. Additional control groups comprised non-CNS neurological diseases (non-CNS; *n* = 18) and orthopedic diseases (OD, *n* = 8).

### Sample collection

Cerebrospinal fluid and serum were obtained from all dogs except the OD group. Sampling was performed after MRI or other diagnostic procedures under general anesthesia. Cerebrospinal fluid was collected via atlanto-occipital (AO; *n* = 121) and lumbar (L; *n* = 4) puncture. Standard CSF analyses were performed within 30 minutes. Cases with incomplete CSF evaluation, iatrogenic blood contamination, and dogs that had already received corticosteroid medication for the presenting condition before referral were excluded.

Blood samples were collected either concurrently with CSF sampling or, in 3 cases where CSF was collected during emergency hours, within 12-24 hours; in these dogs, no corticosteroid treatment was administered during this interval. Both CSF and serum samples were frozen at −80 °C within 1 hour of collection.

### Multiplex immunoassay for cytokine measurement

Interferon-γ, TNF-α, IL-2, IL-6, IL-10, and IL-15 were measured using a multiplex bead-based immunoassay (MILLIPLEX® Canine Cytokine/Chemokine Magnetic Bead Panel—CCYTOMAG-90 K-06 Merck/Sigma, Schaffhausen, Switzerland) on the Luminex™ xMAP™ INTELLIFLEX system with Belysa™ software, per manufacturer’s protocol. Reproducibility was confirmed by repeat measurements.

### Statistical analysis

Statistical analyses were performed using STATA 12. Cytokine data were not normally distributed (Kolmogorov–Smirnov test); non-parametric tests were used. Data are presented as median with interquartile range (IQR); effect estimates as β-coefficient and 95% CIs.

Binomial logistic regression was used to assess the association between cytokine concentrations and MUO diagnosis. The dependent variable was MUO (coded 1) versus IE, intracranial neoplasia, IVDH, and non-CNS (coded 0); OD controls were included in serum analyses only. SRMA was excluded from regression models. Age, sex, and body weight were included as covariates with cluster-robust standard errors (Huber–White estimators). For MUO versus SRMA comparisons, a Mann–Whitney *U* test was applied. Results are presented as ^***^  *P* < .001, ^**^  *P* < .01, ^*^  *P* < .05.

## Results

### Descriptive data

A total of 133 dogs were included and categorized into 7 groups of different clinical conditions: MUO, SRMA, IE, intracranial neoplasia, IVDH, non-CNS, and OD (Table 1).

**Table 1:** Signalment of dogs by disease group.

**Group**	***n***	**Male**	**Female**	**Age in years (median [min; max])**	**Weight in kg (median [min; max])**
**MUO**	23	12	11	4.4 [0.5;10.9]	4.2 [1.6;20.2]
**SRMA**	22	14	8	0.8 [0.1;2.2]	21.3 [3.9;37.5]
**IE**	22	12	10	5.4 [0.8;6.25]	20.7 [4.8;38.8]
**IN**	21	13	8	8.4 [3.8;11.2]	12.0 [3.7;28.8]
**IVDH**	19	12	7	5.4 [3.3;13.6]	12.0 [3.1;31.2]
**Non-CNS (control)**	18	10	8	4.9 [0.5;12.8]	23.6 [4.7;35.0]
**OD (control)**	8	5	3	4.5 [3.0;7.5]	38.0 [32;53.4]
**Overall median**	−	−	−	4.7 [0.5;13.6]	16.3 [1.6;53.4]
**Total**	133	78	55	−	−

This table shows the number of dogs (*n*) across the disease groups: meningoencephalitis of unknown origin (MUO), steroid-responsive meningitis-arteritis (SRMA), idiopathic epilepsy (IE), intracranial neoplasia (IN), intervertebral disc herniation (IVDH), neurologic diseases not involving the CNS (Non-CNS, control), and orthopedic diseases (OD, control). It also presents the distribution of sex (male/female) and provides the median age in years and body weight in kg for each group, with the corresponding minimum and maximum values indicated in square brackets [min;max].

#### Study groups

The MUO group (*n* = 23) consisted of 12 male and 11 female dogs, with a median age of 4.4 years (range: 0.5-10.9 years) and a median body weight of 4.2 kg (range: 1.6-20.2 kg). The median time from onset of clinical signs to presentation was 3 days (range: 1 day to 6 weeks). Based on MRI findings, 20/23 dogs presented with lesions confined to the brain consistent with meningoencephalitis, 2 dogs showed combined brain and spinal cord involvement (meningoencephalomyelitis), and 1 dog presented with isolated spinal cord disease (extensive lumbar meningomyelitis).

The SRMA group (*n* = 22) comprised 14 males and 8 females, with a median age of 0.8 years (range: 0.1-2.2 years) and a median body weight of 21.3 kg (range: 3.9-37.5 kg). The median time from onset of clinical signs to presentation was 5 days (range: 1-28 days). One dog presenting 79 days after onset was excluded due to suspected relapse and recent corticosteroid treatment. Dogs were diagnosed based on cervical pain, meningeal irritation and fever, markedly elevated CRP (median 193.8 mg/L; Table S1), and neutrophilic to mixed CSF pleocytosis.^21^ No abnormalities were detected on cervical radiographs in any dog. MRI was performed in 6/22 dogs and revealed cervical meningopathy, paraspinal myopathy, or both.

The IE group (*n* = 22) comprised 12 males and 10 females, with a median age of 5.4 years (range: 0.8-6.25 years) and a median body weight of 20.7 kg (range: 4.8-38.8 kg). The median time from onset of clinical signs to presentation was 17 weeks (range: 1 day to 4 years). Dogs were diagnosed according to the International Veterinary Epilepsy Task Force (IVETF) Tier II confidence level criteria, including 2 or more unprovoked epileptic seizures at least 24 hours apart, age of onset between 6 months and 6 years, no abnormalities detected on interictal neurological examination, normal blood parameters including preprandial bile acids, no abnormalities detected on brain MRI, and normal CSF analysis.^22^

The intracranial neoplasia group (*n* = 21) included 13 males and 8 females, with a median age of 8.4 years (range: 3.8-11.2 years) and median body weight of 12.0 kg (range: 3.7-28.8 kg). The median time from onset of clinical signs to presentation was 1 month (range: 1 day to 6 months). MRI-based suspected diagnoses included gliomas (*n* = 11), meningiomas (*n* = 3), metastatic neoplasia (*n* = 2), pituitary macroadenoma (*n* = 1), olfactory neuroblastoma (*n* = 1), gliomatosis cerebri/lymphoma (*n* = 1), nerve sheath tumor/lymphoma (*n* = 1), and metastatic process (*n* = 1). Histopathological confirmation was available in 14/21 dogs and included oligodendrogliomas (*n* = 7, WHO grade II–III), meningiomas (*n* = 3), metastatic carcinoma (*n* = 1), histiocytic sarcoma (*n* = 2), and choroid plexus carcinoma (*n* = 1). The MRI-based suspected diagnosis was confirmed histopathologically in 10/14 dogs; in the remaining 4 dogs, histopathology confirmed a different tumor type. Cerebrospinal fluid analysis was within normal limits in 16/21 dogs; in all 5 dogs with mild to moderate pleocytosis, elevated total protein, or both, a confirmed histopathological diagnosis was available.

The IVDH group (*n* = 19) consisted of 12 males and 7 females, with a median age of 5.4 years (range: 3.3-13.6 years) and a median body weight of 12.0 kg (range: 3.1-31.2 kg). The median time from onset of clinical signs to presentation was 7 days (range: 1-42 days). Of 19 dogs, 8 had cervical and 11 thoracolumbar IVDH. Neurological severity was graded according to the Sharp and Wheeler grading system. Cervical cases all presented with grades 1-3 and underwent ventral slot decompression. Thoracolumbar grades ranged from grade 2 to 5; 8 dogs underwent hemilaminectomy, 2 were managed conservatively, and 1 declined surgery. One grade 5 dog had MRI findings suggestive of myelomalacia before surgery, remained grade 5 postoperatively, subsequently developed spinal walking. All dogs were discharged. CSF analysis was within normal limits in all IVDH dogs, with the exception of one cervical IVDH dog with a mildly elevated total nucleated cell count (TNCC) of 15.3 cells/μL.

The non-CNS group (*n* = 18) encompassed 10 males and 8 females, with a median age of 4.9 years (range: 0.5-12.8 years) and a median body weight of 23.6 kg (range: 4.7-35.0 kg). The median time from onset of clinical signs to presentation was 1.5 months [range: 1 day to 2 years]. The group comprised dogs with peripheral neuropathies, neuromuscular diseases, sensory deficits (eg, hearing or visual impairment), ear disease, and behavioral abnormalities without evidence of primary CNS pathology. No abnormalities were detected on brain MRI and CSF analysis in any case.

The OD group (*n* = 8) comprised 5 males and 3 females, with a median age of 4.5 years (range: 3-7.5 years) and the highest median body weight of 38.0 kg (range: 32-53.4 kg). The median time from onset to presentation was 1 month (range: 3 weeks to 3 months; available for 6/8 dogs).

#### CSF total nucleated cell counts and total protein concentrations

Cerebrospinal fluid TNCC and total protein (TP) concentrations varied between disease groups (Table 2). TNCC were available for all dogs in the MUO, SRMA, IE, intracranial neoplasia, IVDH, and non-CNS groups. TP measurements were missing in 6/23 MUO, 2/22 SRMA, 3/21 intracranial neoplasia, 3/19 IVDH, and 1/18 non-CNS dogs, due to emergency sample analysis where only qualitative Pandy testing was available. All IE dogs had complete CSF analyses within reference ranges.

**Table 2:** Median CSF measurements by disease.

**Group**	**TP (g/L [min; max])**	**TNCC (cells/*μ*L [min; max])**
**MUO**	0.3 [0.15; 2.21]	19.3 [0.3; 3754]
**SRMA**	0.7 [0.17; 4.4]	779.0 [30; 8789.3]
**IE**	0.1 [0.07; 0.3]	0.7 [0.3; 4.7]
**IN**	0.3 [0.12; 1.52]	2.7 [0.3; 22.7]
**IVDH**	0.2 [0.11; 0.48]	2.0 [0.7; 15.3]
**Non-CNS (control)**	0.2 [0.07; 0.28]	0.7 [0.3; 2.3]
**OD (control)**	−	−
**Overall**	0.2 [0.07; 4.4]	2.3 [0.3; 8789.3]

This table shows median CSF measurements with minimum and maximum ranges [min;max] by disease: meningoencephalitis of unknown origin (MUO), steroid-responsive meningitis-arteritis (SRMA), idiopathic epilepsy (IE), intracranial neoplasia (IN), intervertebral disc herniation (IVDH), neurologic diseases not involving the CNS (Non-CNS, control), and orthopedic disease (OD, control). Measurements shown include total protein concentration (TP, g/L) and total nucleated cell count (TNCC, cells/μL). No CSF was taken from dogs with primary orthopedic diseases that served as controls.

#### CSF and serum cytokine concentration

Six cytokines (IFN-γ, TNF-α, IL-2, IL-6, IL-10, and IL-15) were measured in CSF and corresponding serum samples across all groups. CSF was not available for the OD group. Distributions are illustrated in Figures 1 and 2.

**Figure 1:**
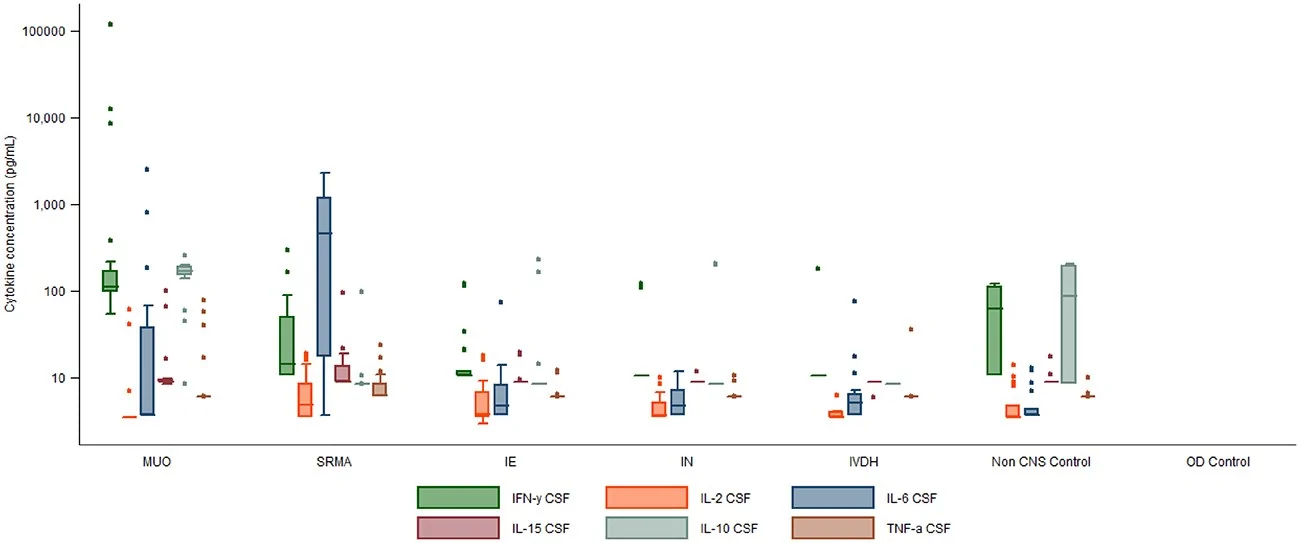
Boxplots of cerebrospinal fluid (CSF) cytokine concentrations (IFN-γ, IL-2, IL-6, IL-15, TNF-α, and IL-10) for dogs diagnosed with meningoencephalitis of unknown origin (MUO), steroid-responsive meningitis-arteritis (SRMA), idiopathic epilepsy (IE), intracranial neoplasia (IN), intervertebral disc herniation (IVDH), and neurologic diseases not involving the central nervous system (non-CNS control). The y-axis represents cytokine concentrations expressed in picograms per milliliter (pg/mL). CSF analysis was not performed in the orthopedic disease group (OD, control) due to the absence of CSF samples.

**Figure 2:**
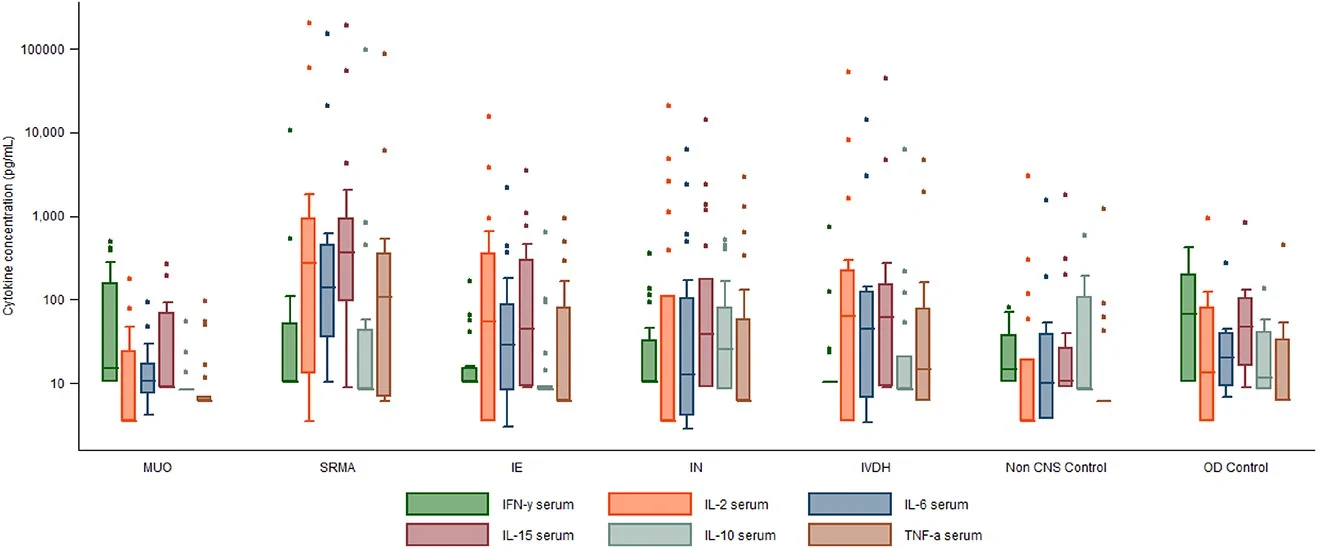
Boxplots of serum cytokine concentrations (IFN-γ, IL-2, IL-6, IL-15, TNF-α, and IL-10) for dogs diagnosed with meningoencephalitis of unknown origin (MUO), steroid-responsive meningitis-arteritis (SRMA), idiopathic epilepsy (IE), intracranial neoplasia (IN), intervertebral disc herniation (IVDH), neurologic diseases not involving the central nervous system (non-CNS, control), and the orthopedic disease control group (OD, control). The y-axis represents cytokine concentrations expressed in picograms per milliliter (pg/mL).

IFN-γ and IL-10 exhibit the greatest discriminatory potential for MUO diagnosis. Median CSF IFN-γ concentration in MUO dogs was 108.4 pg/mL (IQR 25.8) compared to 10.5 pg/mL (IQR 0.0) in the combined comparison group (IE, intracranial neoplasia, IVDH, and non-CNS). SRMA was excluded from the combined comparison group given its potential immunological analogy with MUO. Median CSF IL-10 concentration in MUO dogs was 171.1 pg/mL (IQR 37.0) compared to 8.5 pg/mL (IQR 0.0) in the combined comparison group.

Median CSF concentrations of IL-2, IL-6, IL-15, and TNF-α were comparable between MUO and the combined comparison group, with medians ranging from 3.5 to 9.0 pg/mL (Table S2).

Serum cytokine concentrations did not reveal an apparent pattern distinguishing MUO from the other disease groups (Table S3). Whether CSF and serum cytokines hold discriminatory potential for MUO diagnosis is assessed in detail in the following section.

### Cytokine concentrations in MUO

Based on binomial logistic regression analysis (Table 3.1), dogs with MUO exhibited significantly higher CSF concentrations of IFN-γ (β-coefficient 0.039, 95% CI, 0.021-0.057, *P* < .001), IL-10 (β-coefficient 0.021, 95% CI, 0.011-0.030, *P* < .001), TNF-α (β-coefficient 0.062, 95% CI, 0.023-0.102, *P* < .01), IL-15 (β-coefficient 0.080, 95% CI, 0.012-0.148, *P* < .05) and IL-2 (β-coefficient 0.055, 95% CI, 0.005-0.104, *P* < .05), compared to dogs diagnosed with IE, intracranial neoplasia, IVDH, and non-CNS diseases. For example, each 1 pg/mL increase in CSF IFN-γ is associated with a 0.039 unit increase on the log odds of MUO diagnosis. IL-6 did not show a difference between MUO and the other disease groups.

**Table 3.1:** Regression of CSF cytokine levels in dogs with MUO.

**Dependent: MUO**	**(1)**	**(2)**	**(3)**	**(4)**	**(5)**	**(6)**
**IFN-γ**	0.039^c^	−	−	−	−	−
	**(0.0000)**					
**IL-2**	−	0.055^a^	−	−	−	−
		(0.0309)				
**IL-6**	−	−	0.028	−	−	−
			(0.0866)			
**IL-15**	−	−	−	0.080^a^	−	−
				(0.0206)		
**IL-10**	−	−	−	−	0.021^c^	−
					(0.0000)	
**TNF-α**	−	−	−	−	−	0.062^b^
						(0.0019)
**age**	−0.038	−0.109	−0.125	−0.103	−0.018	−0.126
	(0.7754)	(0.2663)	(0.2367)	(0.2930)	(0.8935)	(0.2192)
**female**	0.580	0.539	0.278	0.639	1.077	0.564
	(0.4519)	(0.3353)	(0.6282)	(0.2701)	(0.1492)	(0.3233)
**weight**	−0.130^a^	−0.131^b^	−0.129^b^	−0.136^b^	−0.132^b^	−0.127^b^
	(0.0120)	(0.0040)	(0.0075)	(0.0055)	(0.0054)	(0.0054)
**Method**	Logit	Logit	Logit	Logit	Logit	Logit
**Pseudo R2**	0.576	0.202	0.261	0.233	0.47	0.234
**Observations**	102	102	102	102	102	102

Cluster-robust standard errors in parentheses.

This table provides the results of the binomial logistic regression assessing the association between cytokine levels in CSF and the diagnosis of meningoencephalitis of unknown origin (MUO). The dependent binary variable is MUO and coded 1 if the dog is diagnosed with MUO and 0 otherwise, including dogs diagnosed with idiopathic epilepsy, intracranial neoplasia, intervertebral disc herniation, and neurologic diseases not involving the central nervous system. CSF samples were not available from orthopedic controls and steroid-responsive meningitis-arteritis was not included in the primary analysis. Each column represents a separate logistic regression model including one cerebrospinal fluid cytokine (IFN-γ, IL-2, IL-6, IL-15, IL-10, or TNF-α). Age refers to the dogs’ ages in years. Female indicates the dog’s sex and assumes 1 if female and 0 otherwise. Weight denotes the dogs’ weight in kg. Cluster-robust standard errors are a clustered version of Huber-White estimators and account for potential clustering at the dog level.

a *P* < .05.

b *P* < .01.

c *P* < .001.

Across all 6 models, lower body weight was independently and significantly associated with the diagnosis of MUO (β-coefficients ranging from −0.127 to −0.136, *P* = .004 to *P* = .012), consistent with the known phenotype of small breed dogs being typically affected by this disease.

In contrast, regression analysis in dogs with MUO did not show significant differences in serum cytokine concentrations compared with dogs diagnosed with IE, intracranial neoplasia, IVDH, non-CNS diseases, or OD controls (Table 3.2).

**Table 3.2:** Regression of serum cytokine levels in dogs with MUO.

**Dependent: MUO**	**(1)**	**(2)**	**(3)**	**(4)**	**(5)**	**(6)**
**IFN-γ**	0.003	−	−	−	−	−
	(0.1960)					
**IL-2**	−	−0.007	−	−	−	−
		(0.0880)				
**IL-6**	−	−	−0.013	−	−	−
			(0.0813)			
**IL-15**	−	−	−	−0.004	−	−
				(0.0943)		
**IL-10**	−	−	−	−	−0.037	−
					(0.0601)	
**TNF-α**	−	−	−	−	−	−0.009
						(0.1505)
**age**	−0.122	−0.090	−0.099	−0.093	−0.091	−0.089
	(0.2144)	(0.3423)	(0.3016)	(0.3284)	(0.3431)	(0.3463)
**female**	0.511	0.491	0.376	0.485	0.532	0.421
	(0.3699)	(0.3880)	(0.5067)	(0.3916)	(0.3625)	(0.4540)
**weight**	−0.121^b^	−0.118^b^	−0.118^b^	−0.123^b^	−0.126^c^	−0.123^b^
	(0.0018)	(0.0011)	(0.0014)	(0.0012)	(0.0009)	(0.0011)
**Method**	Logit	Logit	Logit	Logit	Logit	Logit
**Pseudo R2**	0.229	0.274	0.263	0.259	0.286	0.249
**Observations**	110	110	110	110	110	110

Cluster-robust standard errors in parentheses.

This table provides the results of the binomial logistic regression assessing the association between cytokine levels in serum and the diagnosis of meningoencephalitis of unknown origin (MUO). The dependent binary variable is MUO and coded 1 if the dog is diagnosed with MUO and 0 otherwise, including dogs diagnosed with idiopathic epilepsy, intracranial neoplasia, intervertebral disc herniation, neurologic diseases not involving the central nervous system, and orthopedic diseases. Steroid-responsive meningitis-arteritis was not included in the primary analysis. Each column represents a separate logistic regression model, including one serum cytokine (IFN-γ, IL-2, IL-6, IL-15, IL-10, or TNF-α). Age refers to the dogs’ ages in years. Female indicates the dog’s sex and assumes 1 if female and 0 otherwise. Weight denotes the dogs’ body weight in kg. Cluster-robust standard errors are a clustered version of Huber-White estimators and account for potential clustering at the dog level.

a *P* < .05.

b *P* < .01.

c *P* < .001.

As SRMA represents another inflammatory, non-infectious CNS disease with potential immunological analogy with MUO, SRMA was not included in the binomial logistic regression models. As an unreported robustness analysis, the regression model was additionally repeated including SRMA cases, which did not substantially change the results. In exploratory analyses comparing MUO against each individual comparison group separately, CSF IFN-γ and IL-15 showed the most consistent discriminatory pattern, reaching statistical significance against the majority of individual comparison groups. CSF IL-10 and TNF-α also showed significant differences against most groups, whereas IL-2 and IL-6 showed more variable results across individual comparisons, consistent with the reduced statistical power of separate group analyses. Serum cytokines did not show consistent discriminatory patterns in any individual group comparison.

### Differences in cytokine profiles of MUO and SRMA

To compare cytokine profiles between MUO and SRMA, two-sample Wilcoxon rank-sum (Mann–Whitney *U*) tests were performed for each cytokine in CSF and serum.

Distinct cytokine profiles were observed between MUO and SRMA. In CSF, IFN-γ and IL-10 concentrations were significantly higher in MUO compared to SRMA (both *P* < .001), whereas IL-6 (*P* < .001) and IL-2 (*P* = .011) were significantly higher in SRMA. CSF IL-15 (*P* = .317) and TNF-α (*P* = .093) did not differ between groups (Figure 3; Table S4). In serum, the pattern shifted markedly. IL-2, IL-6, IL-15, TNF-α, and IL-10 were all significantly higher in SRMA compared to MUO (all *P* ≤ .012), consistent with the pronounced systemic inflammatory response characteristic of SRMA (Figure 4; Table S5). Serum IFN-γ did not differ between the 2 groups (*P* = .234).

**Figure 3:**
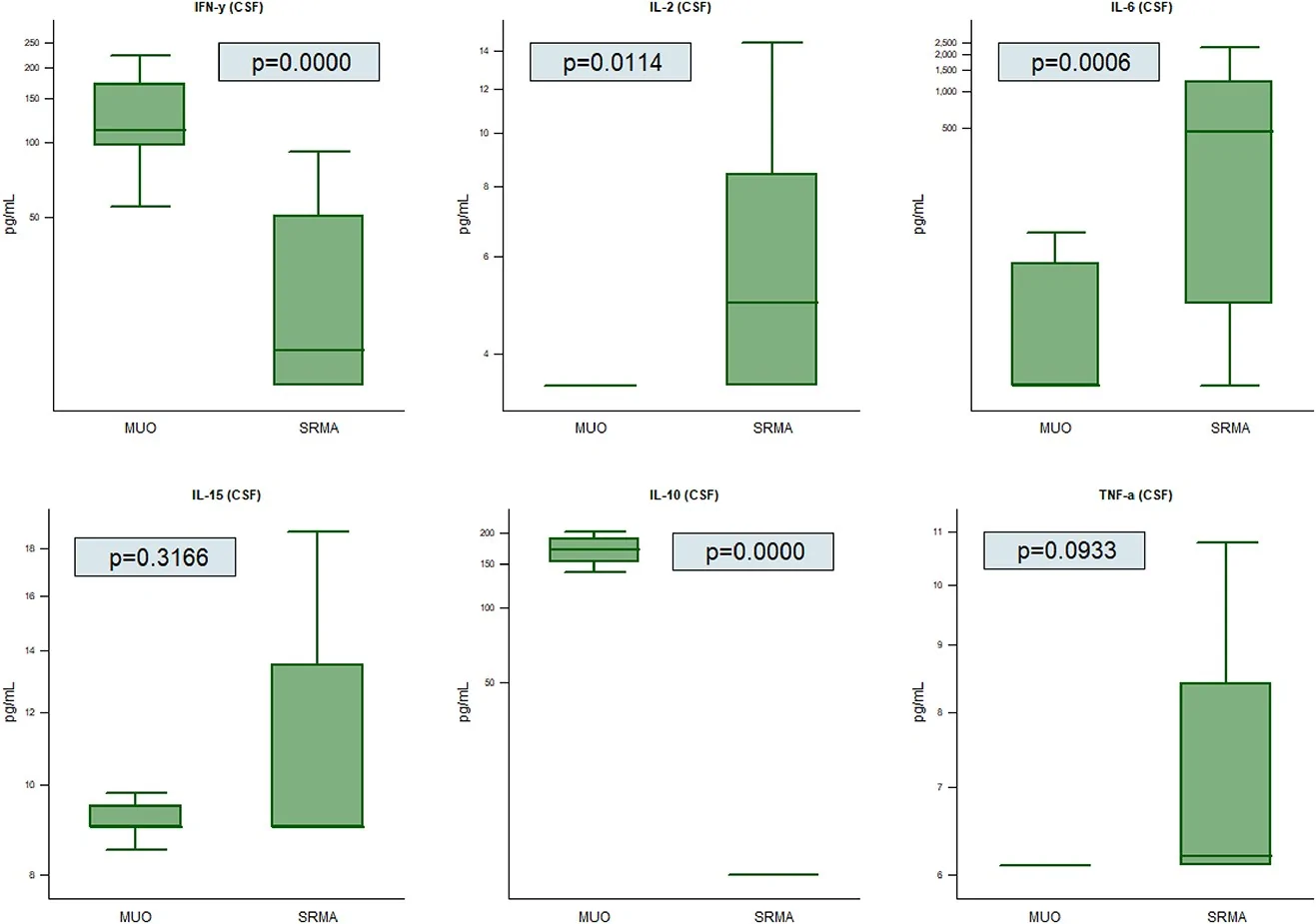
Boxplots of cytokine concentrations of IFN-γ, IL-2, IL-6, IL-15, IL-10, and TNF-α in cerebrospinal fluid (CSF) in dogs with meningoencephalitis of unknown origin (MUO) and steroid-responsive meningitis-arteritis (SRMA). The y-axis represents cytokine concentrations of each cytokine expressed in picograms per milliliter (pg/mL). *P*-values refer to the difference in cytokine levels between MUO and SRMA dogs as a result of the two-sample Wilcoxon rank-sum (Mann–Whitney *U*) test. Boxplots show the median (line), interquartile range (box; 25th-75th percentiles), and minimum–maximum values (whiskers).

**Figure 4:**
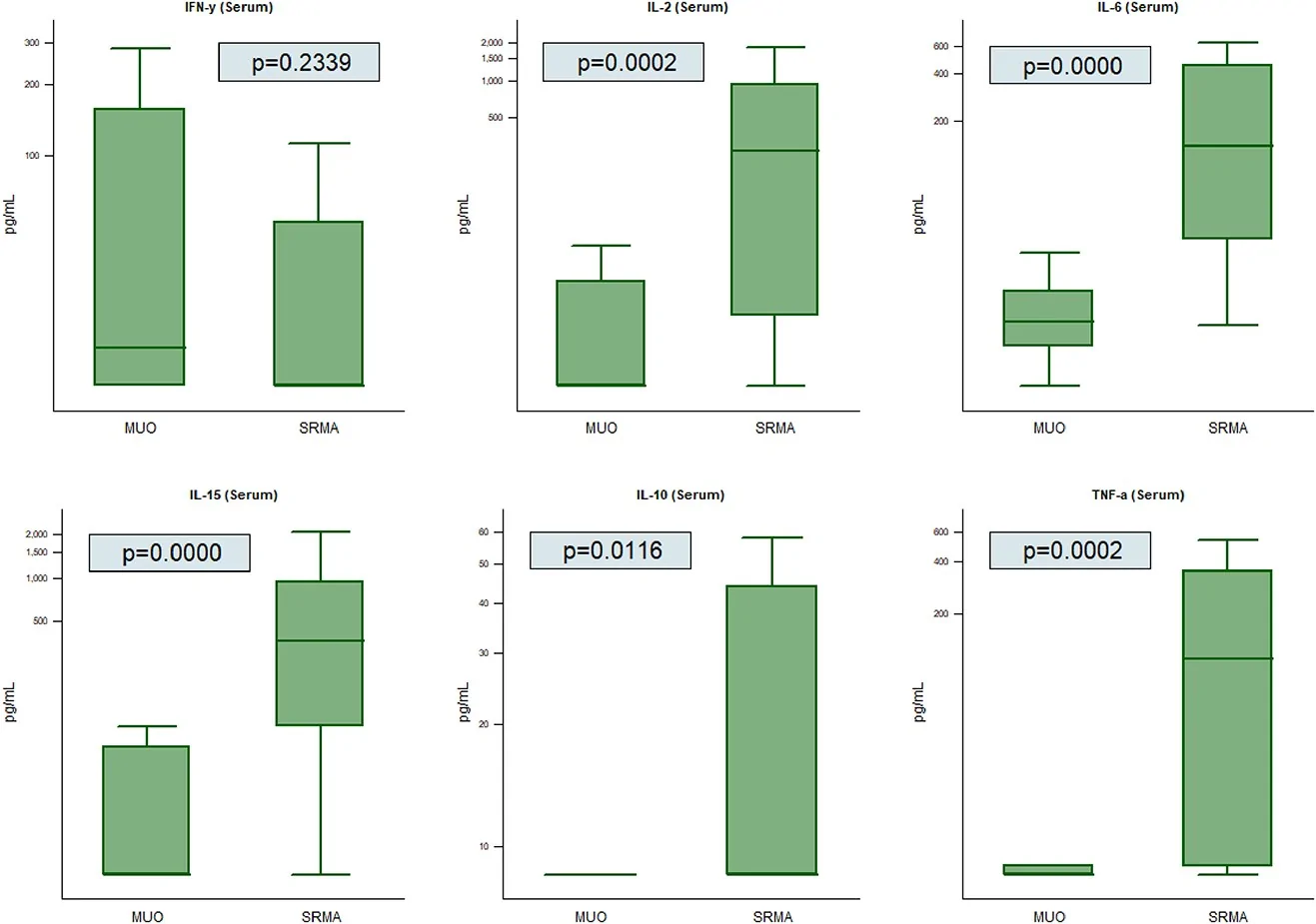
Boxplots of cytokine concentrations of IFN-γ, IL-2, IL-6, IL-15, IL-10 and TNF-α in serum of dogs with meningoencephalitis of unknown origin (MUO) and steroid-responsive meningitis-arteritis (SRMA) groups. The y-axis represents cytokine concentrations of each cytokine expressed in picograms per milliliter (pg/mL). *P*-values refer to the difference in cytokine levels between MUO and SRMA dogs as a result of the two-sample Wilcoxon rank-sum (Mann–Whitney *U*) test. Boxplots show the median (line), interquartile range (box; 25th-75th percentiles), and minimum–maximum values (whiskers).

## Discussion

All 6 cytokines were detectable in paired CSF and serum samples across all disease categories, demonstrating the feasibility of multiplex cytokine profiling in clinical neuroinflammatory diseases of dogs. However, high costs and limited practicality may currently restrict implementation outside specialized research environments. The lack of a definitive diagnostic test for MUO reflects the complex pathophysiology and highlights the challenge of identifying suitable biomarkers for diagnosis, disease monitoring, or treatment stratification.

### Cytokine profile expands current understanding of MUO pathogenesis

Meningoencephalitis of unknown origin was associated with markedly higher CSF concentrations of IFN-γ and IL-10, higher concentrations of TNF-α, and moderately higher concentrations of IL-15 and IL-2 when compared to IE, intracranial neoplasia, IVDH, and non-CNS neurological diseases.

Particularly, high CSF concentrations of IFN-γ and TNF-α in MUO suggest a broader pro-inflammatory cytokine milieu that remains incompletely defined.^2^ Elevated CSF IFN-γ in MUO supports a T helper 1–associated (Th1) mediated response in MUO pathogenesis, consistent with prior reports of IFN-γ upregulation in NME lesions.^7^ Notably, studies in MS have shown that IFN-γ can also exert protective or regulatory functions under certain conditions, highlighting the complexity of its role in neuroinflammation. Whether a similar dual role exists in MUO remains unclear, particularly given the absence of longitudinal data across disease progression. The parallel elevation of TNF-α further emphasizes the contribution of pro-inflammatory pathways, mirroring observations in MS where both IFN-γ and TNF-α are consistently higher in CSF and CNS lesions and drive neuroinflammation.^13^^,^^14^^,^^23^

Byron et al. detected IFN-γ, TNF-α, IL-2, and IL-15 exclusively in CSF of MUO dogs compared to epilepsy and neoplasia.^5^ The present study supports and extends these findings, additionally identifying elevated IL-10 in MUO versus IE, intracranial neoplasia, IVDH and non-CNS diseases.

While the present study supports a CNS-compartmentalized, predominantly Th1-driven immune response in MUO, accumulating evidence suggests that the immunopathogenesis is multifaceted. Subtype-specific differences may partly explain this heterogeneity: while NME is characterized by a predominantly T-cell-driven response with IFN-γ production contributing to neuronal necrosis, GME shows a more mixed pattern including IL-17 and IL-4, suggesting concurrent Th2 and Th17 components.^24^

This compartmentalization between CNS and peripheral immune responses is further supported by flow cytometric analysis of peripheral blood mononuclear cells in dogs with MUO, which revealed significantly more CD4+ IL-17-producing T-cells but fewer IFN-γ-producing CD4+ T-cells compared to healthy controls, suggesting concurrent Th17 activity in the systemic compartment alongside reduced peripheral Th1 responses.^3^ Combined with the absence of elevated serum IFN-γ and the markedly elevated CSF IFN-γ concentrations observed in the present study, this suggests preferential recruitment of IFN-γ-producing T-cells into the CNS rather than the peripheral circulation.

Adding further complexity, Church et al. demonstrated meningeal B-cell accumulation with local expression of the chemokines CXCL13 and CCL21 in dogs with GME, suggesting the formation of tertiary lymphoid organ-like structures within the leptomeninges.^25^ These structures may provide an additional site for local antigen presentation and B-cell activation, potentially sustaining the intrathecal inflammatory response in MUO. Taken together, these findings suggest that Th1, Th17, and B-cell-mediated mechanisms may operate concurrently in different anatomical compartments in MUO, potentially explaining the heterogeneity observed across studies.

High CSF IL-10 in MUO contrasts with the otherwise pro-inflammatory profile, likely representing reactive regulation in response to Th1 activity (IFN-γ, TNF-α), suggesting an attempt of the immune system to counteract inflammation. Similar findings have been reported in MS, where CSF IL-10 is elevated compared to controls.^26^ While sometimes linked to disease activity, higher IL-10 has also been associated with less disability progression and better-preserved brain structures,^27^^,^^28^ suggesting potential neuroprotective effects. Whether elevated IL-10 implies slower progression in MUO warrants further investigation.

The distinct intrathecal cytokine profile in MUO indicates a Th1-skewed pro-inflammatory response accompanied by limited compensatory regulation through IL-10. The elevation of IL-15 and IL-2 further indicates active T-cell proliferation and survival, pointing to an adaptive immune response that may contribute to chronicity of the disease. Importantly, these alterations were confined to the CSF and not reflected in serum, underscoring the CNS-specific nature of the immune response in MUO.

Serum cytokine concentrations did not differ between MUO and the other groups, suggesting that systemic markers may not reliably capture intrathecal immune activity. Nevertheless, immunosignature assays have distinguished dogs with MUO from intracranial neoplasia and healthy controls based on antibody-binding patterns.^29^ Thus, while the serum cytokine assays in the present study appeared to lack diagnostic utility for MUO, antibody-based profiling may offer valuable complementary tools.

Although the present study included comparatively large groups of animals, further validation in expanded cohorts is essential, and more detailed investigations are necessary to better differentiate between MUO subtypes, including necrotizing leukoencephalitis (NLE), GME, and NME, to clarify their diagnostic and prognostic value.

In addition, our regression analyses consistently identified lower body weight as significantly associated with MUO, reflecting the well-established breed predisposition for toy and small-breed dogs and confirming that the study sample is representative of the typical MUO signalment.

Intracranial neoplasia, and in particular gliomas, represent important differential diagnoses for MUO, as inflammatory CNS lesions, especially granulomas including the focal form of GME, may present with overlapping MRI features indistinguishable from glioma without histopathological confirmation.^30^ Notably, in the present study, dogs with histopathologically confirmed or MRI-suspected gliomas showed no CSF abnormalities and no elevated CSF cytokine concentrations. Although CSF analysis can be normal in up to 22% of MUO cases,^1^ the absence of elevated CSF cytokine concentrations in these dogs suggests that cytokine profiling may provide additional discriminatory value in differentiating glioma from MUO, complementing conventional CSF analysis. Furthermore, histiocytic sarcoma affecting the CNS is associated with pronounced CSF inflammation, often resulting in markedly elevated TNCC that may mimic inflammatory CNS disease.^31^ One dog with confirmed histiocytic sarcoma in the present study showed mild elevations in CSF IL-2, IL-6, IL-15, and TNF-α concentrations alongside moderate mixed pleocytosis, which may complicate antemortem differentiation from inflammatory CNS diseases such as MUO before histopathological confirmation. All dogs with CSF pleocytosis had histopathological confirmation of neoplasia, supporting the diagnostic validity of this group.

Intervertebral disc herniation triggers pro-inflammatory immune activation in the spinal cord,^32^ which can independently induce cytokine release and complicate interpretation of cytokine patterns. Intervertebral disc herniation dogs presented across neurological severities (grades 1-5), including 1 dog with MRI findings suggestive of myelomalacia that recovered to spinal walking, indicating a non-progressive or focal form rather than true progressive myelomalacia. Although more severe cases could theoretically exhibit higher intrathecal cytokine concentrations reflecting greater inflammatory responses, CSF findings were largely within normal limits across the IVDH group, with only 1 dog showing a mildly elevated nucleated cell count as detailed in the Results, suggesting that cytokine levels did not reflect clinically relevant neuroinflammation regardless of injury severity.

### Distinction of cytokine profiles in MUO and SRMA

Meningoencephalitis of unknown origin and SRMA are both inflammatory, non-infectious diseases affecting the CNS, the leptomeninges and associated arteries, respectively. While MUO and SRMA are clinically and diagnostically readily distinguishable, a direct comparison of cytokine concentrations in paired CSF and serum samples represents a valuable approach to explore disease-specific immune activation patterns. Cytokine concentrations were measurable in all CSF and serum samples from both groups, supporting the feasibility of comparative cytokine profiling.

The significantly higher CSF concentrations of IFN-γ and IL-10 in MUO are consistent with a Th1-skewed response accompanied by compensatory regulation. In contrast, SRMA showed significantly higher IL-6 and IL-2 levels in CSF, highlighting the central role of this cytokine in SRMA pathogenesis.

In serum, cytokine analyses in dogs with SRMA exhibited higher median concentrations of IL-2, IL-6, IL-10, IL-15, and TNF-α compared to all other disease groups and controls, highlighting disease-specific systemic immune activation. The finding of higher IL-6 in both CSF and serum in SRMA is in line with findings of various studies that identify IL-6 as a key mediator in SRMA, driving systemic inflammation, neutrophil recruitment, and disruption of the blood–brain barrier.^6^^,^^16^^,^^19^^,^^21^^,^^33^

While earlier work suggested a Th2/Th17 profile with downregulated Th1 cytokines such as IL-2 and IFN-γ,^18^^,^^20^ the current study revealed markedly higher serum IL-2 and IL-15 concentrations. Elevated IL-15, not previously reported in SRMA, is biologically plausible given its role in T- and NK-cell activation and may represent an upstream driver of excessive T-cell proliferation in genetically predisposed breeds.^34^ These findings point to IL-15 as a novel marker and potential pathogenic factor in SRMA. Although early studies failed to detect TNF-α activity in SRMA serum, our data suggest that TNF-α contributes to the broader pro-inflammatory cytokine storm.^16^^,^^19^ The absence of serum cytokine changes in MUO highlights CNS-compartmentalization of inflammation, consistent with normal acute phase proteins in MUO.

In contrast, SRMA was associated with a systemic cytokine storm in serum, dominated by IL-6, IL-2, IL-15, and TNF-α, and accompanied by marked leukocytosis and elevated CRP. These findings reaffirm the pivotal role of IL-6 in SRMA pathogenesis and further implicate IL-15 and TNF-α as potential novel contributors to the dysregulated immune response. Thus, while serum biomarkers appear to have limited diagnostic value in MUO, they provide clinically useful information in SRMA, with CRP serving as a particularly robust marker of systemic inflammation.

## Conclusions

In this prospective observational study, multiplex bead-based cytokine profiling was feasible in paired CSF and serum samples across a broad spectrum of neurological diseases of dogs and controls. Dogs with MUO exhibited a distinct intrathecal cytokine pattern characterized by significantly higher CSF concentrations of IFN-γ, IL-10, and TNF-α, together with higher IL-15 and IL-2, when compared to dogs diagnosed with IE, intracranial neoplasia, IVDH, and non-CNS diseases, supporting a Th1-skewed, CNS-compartmentalized immune response.

Meningoencephalitis of unknown origin and SRMA displayed clearly divergent cytokine profiles: MUO was characterized by higher CSF IFN-γ and IL-10 concentrations, whereas SRMA showed higher IL-6 in CSF and pronounced systemic cytokine elevations in serum (IL-2, IL-6, IL-15, and TNF-α), consistent with robust peripheral inflammatory activation. Accordingly, serum cytokines and acute-phase markers appeared of limited diagnostic utility for MUO but provided clinically meaningful information in SRMA.

Based on these findings, selected CSF cytokines may represent promising components of future biomarker panels to advance the understanding of MUO immunopathogenesis and support future diagnostic and therapeutic research. However, further validation in larger cohorts, targeted evaluation across MUO subtypes (NLE, GME, NME), and assessment of their prognostic relevance under treatment are required before clinical implementation.

### Limitations

The sample size within each disease group remained modest compared to studies in human medicine, which restricts statistical power and may limit the generalizability of the results. Nevertheless, the overall number of cases included in the present study was relatively large in canine neurology.

Although samples were stored at −80 °C, which is considered the standard for long-term cytokine preservation, the 5-year sample collection period raises the possibility of differential cytokine degradation. Some cytokines may be more susceptible to freeze–thaw degradation or long-term storage effects than others, which could have introduced variability in measured concentrations across the collection period.

The timing of sample collection in relation to the onset of clinical signs could influence cytokine concentrations in CSF and serum. In our cohort, MUO and SRMA cases were presented much earlier after onset of signs (median within 3 and 5 days, respectively) compared to dogs with intracranial neoplasia (median 1 month) or IE (median 4 months). As cytokine responses are dynamic and may change along the disease course, more acute disease entities such as in MUO or SRMA could reflect higher intrathecal cytokine concentrations, whereas subacute or chronic stages (eg, IE or neoplasia) may already show normalization or redistribution of inflammatory signaling. It is also conceivable that differences in the temporal relationship between disease onset and clinical presentation exist between MUO and SRMA: clinical signs in SRMA, as a systemic inflammatory disease, may manifest and be recognized earlier, whereas in MUO the inflammatory process may already be ongoing for a longer period before overt neurological signs become apparent. This introduces a potential time-dependent bias that may have influenced inter-group comparisons.

In dogs with IE, the temporal relationship between seizure activity and CSF sampling was not systematically documented. Seizures are known to trigger transient systemic and intrathecal increases in cytokines.^35^ Consequently, we cannot exclude the possibility that IE may involve short-lived neuroinflammatory responses, and the heterogeneous interval between the first seizure and MRI/CSF collection could have led to underestimation of such effects.

Within the IVDH group, neurological severity varied considerably (grades 1-5), and the potential influence of injury severity and associated spinal cord inflammation on cytokine concentrations was not formally assessed.

Some dogs had received anti-inflammatory pretreatment, whose effect on cytokine levels was not systematically assessed. Recent treatment with non-steroidal anti-inflammatory drugs (NSAIDs) was not formally recorded as an exclusion criterion, and many dogs were referred from primary care veterinarians and may have received NSAIDs before referral, the nature and duration of which were not always fully documented. This may have introduced variability in cytokine concentrations across groups, with the exception of the IE group in which NSAIDs were generally not indicated. Variation in MRI timing and anesthesia before CSF collection could further have introduced kinetic variability.

Further, as a cross-sectional study, our analysis was limited to single time-point comparisons without longitudinal follow-up. Future work should include adjustment for the interval between onset of signs and sampling, stratification of IE cases by peri-/postictal status and time since last seizure, and longitudinal serial sampling under treatment to better capture cytokine kinetics and assess their potential as biomarkers for disease monitoring and prognosis.

Finally, no distinction was made between MUO subtypes (GME, NME, NLE), which may exhibit distinct cytokine patterns but cannot be reliably differentiated antemortem. It remains conceivable, however, that cytokine profiles may vary between these subgroups, which could not be addressed in the present study.

Furthermore, the comparison groups in the present study did not include dogs with infectious CNS diseases, which represent clinically relevant differential diagnoses for MUO. The diagnostic relevance of CSF cytokine profiling will only become apparent once comparisons are extended to other inflammatory CNS conditions, particularly infectious encephalitis.

## Supplementary Material

Supplementary_Material_aalag248

## Data Availability

The data supporting the findings of this study are available from the corresponding author upon reasonable request.

## Off-label antimicrobial declaration

Authors declare no off-label use of antimicrobials.

## Institutional animal care and use committee or other approval declaration

The study protocol was reviewed and approved by the Ethical Committee of the Veterinary Service, Canton of Bern (BE121/2020). Written informed consent was obtained from all dog owners before participation.

## Appendix

### Further Background

A definitive diagnosis of MUO requires histopathological confirmation, which is often not feasible due to the invasiveness and costs of brain biopsy. This limitation has prompted increasing interest in minimally invasive biomarkers, with cytokines representing promising candidates given their central role in regulating immune responses.^4^^,^^26^

Cytokines are small, secreted proteins that regulate immune responses by mediating cell-to-cell communication and orchestrating both local and systemic inflammatory processes. They can be broadly categorized as proinflammatory or anti-inflammatory, and certain subsets are further defined by their role in T-helper cell responses.

In our prospective study, we analyzed a panel of 6 cytokines (IL-10, IL-15, IL-2, IL-6, IFN-γ, and TNF-α). This selection was determined by the availability of the assay kit and guided by existing evidence from canine studies as well as data from human MS research.

### Materials and methods

This prospective observational study included client-owned dogs that were presented to the Neurology Division, Vetsuisse Faculty, University of Bern, due to neurological deficits between 2018 and 2023. Signalment, anamnesis, and clinical history were systematically recorded for all cases, followed by a comprehensive clinical and neurological examination. Routine hematological and biochemical blood tests were performed in all dogs except those in the OD group, where only basic biochemical blood measurements were available. In all MUO cases, infectious causes were evaluated by serologic testing for toxoplasmosis and neosporosis. Additionally, a screening test for *Anaplasma* spp., *Ehrlichia* spp., *Borrelia burgdorferi*, and *Dirofilaria immitis* was performed in 17/23 dogs, and an Angiostrongylus SNAP test in 3/23 dogs. PCR testing for canine distemper virus in CSF was performed in 4/23 dogs.

**Appendix Table 1:** Breed distribution. This table provides the breed distribution for all dogs included in the study, across the following disease groups: meningoencephalitis of unknown origin (MUO), steroid-responsive meningitis-arteritis (SRMA), idiopathic epilepsy (IE), intracranial neoplasia (IN), intervertebral disc herniation (IVDH), neurological diseases not involving the central nervous system (non-CNS, control), and orthopedic diseases (OD, control).

Group	Breeds
**MUO** **(*n* = 23)**	Chihuahua (*n* = 7), Yorkshire Terrier (*n* = 3), mixed-breed (*n* = 3), Pomeranian (*n* = 2), French Bulldog (*n* = 2), and *n* = 1 each: Cairn Terrier, Pekingese, Bichon Maltese, Australian Shepherd, Bolonka Zwetna, Pug
**SRMA** **(*n* = 22)**	Boxer (*n* = 6), mixed-breed (*n* = 3), Bernese Mountain Dog (*n* = 2), Beagle (*n* = 2), and *n* = 1 each: Golden Retriever, Sabueso Español, German Wirehaired Pointer, Lhasa Apso, Irish Terrier, Pyrenean Shepherd, Nova Scotia Duck Tolling Retriever, Briard, Havanese
**IE** **(*n* = 22)**	Mixed-breed (*n* = 5), Border Collie (*n* = 3), Australian Shepherd (*n* = 3), Poodle (*n* = 2), and *n* = 1 each: Staffordshire Bull Terrier, Staffordshire Bull Terrier mix, American Pitbull Terrier, Jack Russell Terrier, Airedale Terrier, Pomeranian, Miniature Pinscher, Gordon Setter, Chihuahua
**IN** **(*n* = 21)**	French Bulldog (*n* = 4), mixed-breed (*n* = 3), and *n* = 1 each: Border Collie, Lhasa Apso, Shih Tzu, Border Terrier, Labrador Retriever, Bolonka Zwetna, Dachshund, Vizsla, American Staffordshire Terrier, Pomeranian, Beagle, Golden Retriever, Boxer, Pug
**IVDH** **(*n* = 19)**	French Bulldog (*n* = 11), Dachshund (*n* = 4), and *n* = 1 each: Shih Tzu, American Staffordshire Terrier, German Shepherd, Staffordshire Bull Terrier
**Non-CNS (control) (*n* = 18)**	Mixed-breed (*n* = 4), Labrador Retriever (*n* = 3), and *n* = 1 each: Lagotto Romagnolo, German Shepherd, Cavalier King Charles Spaniel, Pomeranian, Belgian Malinois, Polish Lowland Sheepdog, White Swiss Shepherd, Basenji, Flat Coated Retriever, Lhasa Apso, Beagle
**OD (control)** **(*n* = 8)**	*n* = 1 each: Australian Shepherd, White Swiss Shepherd, Rottweiler, Boxer, mixed-breed, Transylvanian Scent-Hound, Bernese Mountain Dog, Cane Corso Italiano

Most dogs additionally underwent advanced imaging, including magnetic resonance imaging (MRI) of the central nervous system (CNS), depending on clinical indication and diagnostic feasibility. MRI was performed in 100% of dogs in the MUO, idiopathic epilepsy (IE), intracranial neoplasia, and intervertebral disc herniation groups. In the steroid-responsive meningitis-arteritis (SRMA) group, only about 27% of dogs (6/22) underwent CNS MRI. In the non-CNS group, 94% (17/18) had CNS MRI, whereas none of the OD dogs received CNS MRI, although some may have had MRI of joints such as the stifle. Cerebrospinal fluid analysis was conducted in all dogs except those in the OD group. CSF was collected in lateral recumbency using plain polypropylene tubes. Standard CSF analyses included total protein concentration, total nucleated cell count (TNCC), and cytological differentiation, and were performed within 30 minutes of collection. Serum samples were centrifuged for 10 minutes at 3000 × *g* and 20 °C before freezing.

To ensure robust statistical analysis, the study cohort was expanded. In total, samples from 133 dogs were analyzed, including individuals with meningoencephalomyelitis of unknown origin (MUO, *n* = 23), SRMA (*n* = 22), IE (*n* = 22), intracranial neoplasia (*n* = 21), IVDH (*n* = 19), systemic non-CNS diseases (*n* = 18), and orthopedic diseases (OD, *n* = 8). Cytokine measurements were completed by January 2025 and subsequently subjected to statistical analysis.

CSF and serum samples were centrifuged after collection within 30 minutes, aliquoted, and stored at −80 °C until analysis. Repeat measurements of representative samples confirmed the high reproducibility of the results. The panel catalog number was CCYTOMAG-90 K-06. The Luminex™ xMAP™ INTELLIFLEX system was operated with xPONENT® software version 4.2 (Luminex, Thermo Fisher Scientific, Waltham, MA, USA). Data analysis was carried out using Belysa™ software (Merck/Sigma, Schaffhausen, Switzerland).

In the MUO group, CSF was collected in 23/23 dogs (21 AO, 2 L). Total protein was available for 17/23, and cell counts for 23/23 dogs. Most dogs (18/23) were presented within the first week of onset of clinical signs, 4 within 2-4 weeks, and 1 dog after more than 4 weeks.

In the SRMA group, CSF was collected in 22/22 dogs (21 AO, 1 AO + L). Total protein was available for 20/22, and cell counts for 22/22 dogs. Fourteen of 22 dogs were presented within 7 days, 7 dogs within 14-28 days, and 1 dog after more than 28 days. In the IE group, CSF was collected in 22/22 dogs (all AO). Total protein and cell counts were available for 22/22 dogs. In the IE group, 4 dogs were presented within the first week, 8 within 2-12 weeks, 6 between 3 months and 1 year, and 4 only after more than 1 year.

In the intracranial neoplasia group, CSF was collected in 21/21 dogs (all AO). Total protein was available for 18/21, and cell counts for 21/21 dogs. Seven dogs were presented within the first week of showing clinical signs, 5 within 2-4 weeks, and nine between 1 and 6 months. In the IVDH group, CSF was collected in 19/19 dogs (18 AO, 1 L). Total protein was available for 16/19, and cell counts for 19/19 dogs. Twelve dogs were presented within the first week, 6 within 2-4 weeks (all of which had cervical IVDH), and 1 after more than 4 weeks. In the non-CNS group, 8 dogs were presented within the first month, 7 between 1 and 6 months, 1 between 6 and 12 months, and 2 dogs after more than 1 year. In the OD group, 4 dogs were presented within the first month, 2 after 2 and 3 months, respectively, and for 2 dogs the interval was not documented.

### Additional considerations

The following section summarizes additional considerations relevant to the interpretation of our cytokine findings. Although not directly part of our primary analyses, these points provide a broader context regarding cytokine behavior in other diseases.

SRMA was not included in the binomial logistic regression models to avoid conflating inflammatory disease categories when evaluating cytokine associations with MUO. In addition to the CSF cytokine profile, SRMA is characterized by a pronounced systemic inflammatory response. In line with this systemic IL-6-driven component, the present study showed that the SRMA group displayed markedly higher blood measurements of inflammation. Median leukocyte counts were much higher than in all other groups, driven predominantly by segmented neutrophilia, which is consistent with acute systemic inflammation. Similarly, CRP values were strikingly elevated in SRMA (median 193.8 mg/L), whereas all other groups remained below 10 mg/L. This underlines the value of CRP as a strong marker of systemic inflammation and highlights its diagnostic importance in the blood analysis of SRMA dogs, while confirming previous observations that CRP has limited diagnostic value in MUO.^2^^,^^16^

Beyond MUO and MS, cytokine alterations have also been implicated in other neurologic diseases. For instance, recent studies indicate that epileptic seizures can induce the production of several cytokines that may influence both the pathogenesis and clinical course of epilepsy. In humans, particular emphasis has been placed on IL-1β, IL-6, and TNF-α, all of which have been shown to rise following seizure activity.^36^ These observations underscore that cytokine dysregulation is not specific to immune-mediated diseases but can occur secondary to neuronal hyperexcitability.

Finally, even non-neurological disorders may contribute to cytokine alterations. A noteworthy observation in our study was the elevation of serum IFN-γ in OD with cruciate ligament rupture. This increase is most plausibly related to immune activation from tissue injury and joint inflammation. Although routine bloodwork did not reveal overt abnormalities, IFN-γ may reflect subtle inflammatory processes, consistent with previous reports demonstrating upregulation of IFN-γ, IL-8, and IL-10 in synovial fluid of cruciate ligament rupture cases.^37^

## Abbreviations

CNS: central nervous system
CSF: cerebrospinal fluid
IE: idiopathic epilepsy
IFN-γ: interferon-γ
IL-2: interleukin-2
IL-6: interleukin-6
IL-10: interleukin-10
IL-15: interleukin-15
IN: intracranial neoplasia
IVDH: intervertebral disc herniation
MUO: meningoencephalitis of unknown origin
OD: orthopedic diseases
SRMA: steroid-responsive meningitis-arteritis
Th1: T-helper 1 cell
TNCC: total nucleated cell count
TNF-α: tumor necrosis factor-α
TP: total protein
