# Supplementary material for: Comparative cytokine profiling in cerebrospinal fluid and serum of dogs with meningoencephalitis of unknown origin
Source: J Vet Intern Med. 2026 Nov 10;40(6):aalag248. doi: 10.1093/jvimsj/aalag248 (PMC13750368; doi:10.1093/jvimsj/aalag248)
Supplement: Supplementary file 1 [file supplementary_material_aalag248.docx]

# **SUPPLEMENTARY MATERIAL**

| Table S1: Selected median blood measurements by disease | | | | | | | | |
| --- | --- | --- | --- | --- | --- | --- | --- | --- |
| *This table shows selected median blood measurements by disease group: meningoencephalitis of unknown origin (MUO), steroid-responsive meningitis-arteritis (SRMA), idiopathic epilepsy (IE), intracranial neoplasia (IN), intervertebral disc herniation (IVDH), neurologic diseases not involving the CNS (Non-CNS, control), and orthopedic diseases group (OD, control). Abbreviations: WBC = white blood cell count; NEU = neutrophil count; LYMPH = lymphocyte count; MONO = monocyte count; TP = total protein; ALB = albumin; GLOB = globulin; CRP = C-reactive protein.* | | | | | | | | |
| Group | WBC  [min; max]  *x10^9^/L* | NEU  [min; max]  *x10^9^/L* | LYMPH  [min; max]  *x10^9^/L* | MONO  [min; max]  *x10^9^/L* | TP  [min; max]  *g/L* | ALB  [min; max]  *g/L* | GLOB  [min; max]  *glL* | CRP  [min; max]  *mg/L* |
| MUO | 9.0  [3.6; 28.2] | 7.5  [3.1; 25.5] | 1.2  [0.4; 2.9] | 0.5  [0.2; 1.6] | 64.4  [53.9; 82.7] | 35.4  [28.5; 43.7] | 26.1  [18.9; 39.0] | 3.3  [0.0; 136.0] |
| SRMA | 19.0  [12.0; 36.0] | 15.5  [7.6; 27.1] | 2.8  [0.9; 7.6] | 1.9  [0.3; 6.0] | 67.0  [58.0; 82.0] | 31.8  [22.4; 37.4] | 35.0  [25.0; 49.0] | 193.8  [21.0; 345.2] |
| IE | 7.8  [3.8; 10.7] | 6.9  [3.5; 9.0] | 1.5  [0.8; 4.9] | 0.4  [0.1; 1.2] | 60.4  [51.6; 71.0] | 34.9  [27.0; 42.1] | 25.8  [20.0; 42.0] | 6.5  [0.0; 44.2] |
| IN | 9.1  [3.0; 25.6] | 7.2  [2.2; 22.8] | 1.2  [0.4; 2.4] | 0.5  [0.1; 1.9] | 65.0  [47.9; 72.0] | 36.3  [24.5; 41.6] | 27.9  [23.3; 38.0] | 4.6  [0.0; 66.8] |
| IVDH | 7.6  [3.9; 17.9] | 6.2  [0.0; 13.1] | 1.4  [0.7; 2.9] | 0.3  [0.2; 1.2] | 67.2  [51.4; 79.7] | 37.1  [30.9; 48.9] | 28.4  [20.5; 37.0] | 1.6  [0.0; 4.6] |
| Non-CNS (control) | 9.1  [5.8; 35.0] | 6.6  [3.0; 31.2] | 2.3  [1.0; 3.8] | 0.4  [0.0; 1.0] | 66.0  [51.8; 90.5] | 35.4  [16.7; 39.5] | 32.5  [19.9; 68.1] | 6.2  [1.4; 129.8] |
| OD (control) | 8.1  [5.5; 13.3] | 6.1  [6.1; 6.1] | 3.0  [3.0; 3.0] | 0.8  [0.8; 0.8] | 61.1  [56.2; 67.4] | 37.3  [29.2; 38.7] | 26.2  [22.7; 37.8] | 3.2  [1.5; 6.3] |
| Overall | 9.2  [3.0; 36.0] | 7.8  [0.0; 31.2] | 1.6  [0.4; 7.6] | 0.6  [0.0; 6.0] | 65.0  [47.9; 90.5] | 35.3  [16.7; 48.9] | 27.3  [18.9; 68.1] | 6.9  [0.0; 345.2] |

| Table S2: Summary statistics of CSF cytokine associations in MUO | | | | | | | | |
| --- | --- | --- | --- | --- | --- | --- | --- | --- |
| *This table summarizes the descriptive statistics of the CSF cytokine measurements in dogs with meningoencephalitis of unknown origin (MUO) versus the comparison group (Panel A) and the results of the binomial logistic regression assessing the association between cytokine levels in CSF and MUO diagnosis (Panel B). The comparison group comprises dogs diagnosed with idiopathic epilepsy (IE), intracranial neoplasia (IN), intervertebral disc herniation (IVDH), and neurologic diseases not involving the central nervous system (non-CNS). CSF samples were not available from orthopedic disease (OD) controls. Panel A reports medians and interquartile ranges (IQR) for each cytokine. Panel B presents the results of separate binomial logistic regression models, each including one CSF cytokine as predictor. The dependent variable is coded 1 for MUO and 0 for the comparison group. β-coefficients, Wald statistics (z), and p-values are reported. Outliers were excluded using Tukey's 1.5 × IQR rule, with values below Q1 − 1.5 × IQR or above Q3 + 1.5 × IQR considered outliers.* | | | | | | | | |
| CSF Cytokine | Panel A | | | |  | Panel B | | |
|  | Median | | IQR | |  | β-coefficient | Wald  statistic (z) | *p*-value |
|  | MUO | Comparison | MUO | Comparison |  |  |  |  |
| IFN-γ | 108.4 | 10.5 | 25.8 | 0.0 |  | 0.039*** | 4.24 | 0.0000 |
| IL-2 | 3.5 | 3.5 | 0.0 | 1.1 |  | 0.055* | 2.16 | 0.0309 |
| IL-6 | 3.7 | 3.9 | 0.2 | 3.4 |  | 0.028 | 1.71 | 0.0866 |
| IL-15 | 9.0 | 9.0 | 0.0 | 0.0 |  | 0.080* | 2.31 | 0.0206 |
| IL-10 | 171.1 | 8.5 | 37.0 | 0.0 |  | 0.021*** | 4.27 | 0.0000 |
| TNF-α | 6.1 | 6.1 | 0.0 | 0.0 |  | 0.062** | 3.10 | 0.0019 |
| *** *p<0.001*, ** *p<0.01*, * *p<0.05* | | | | | | | | |

| Table S3: Summary statistics of serum cytokine associations in MUO | | | | | | | | |
| --- | --- | --- | --- | --- | --- | --- | --- | --- |
| *This table summarizes the descriptive statistics of the serum cytokine measurements in dogs with meningoencephalitis of unknown origin (MUO) versus the comparison group (Panel A) and the results of the binomial logistic regression assessing the association between serum cytokine levels and MUO diagnosis (Panel B). The comparison group comprises dogs diagnosed with idiopathic epilepsy (IE), intracranial neoplasia (IN), intervertebral disc herniation (IVDH), neurologic diseases not involving the central nervous system (non-CNS), and orthopedic disease (OD) controls.* *Panel A reports medians and interquartile ranges (IQR) for each cytokine. Panel B presents the results of separate binomial logistic regression models, each including one serum cytokine as predictor. The dependent variable is coded 1 for MUO and 0 for the comparison group. β-coefficients, Wald statistics (z), and p-values are reported.* *Outliers were excluded using Tukey's 1.5 × IQR rule, with values below Q1 − 1.5 × IQR or above Q3 + 1.5 × IQR considered outliers.* | | | | | | | | |
| Serum Cytokine | Panel A | | | |  | Panel B | | |
|  | Median | | IQR | |  | β-coefficient | Wald  statistic (z) | *p*-value |
|  | MUO | Comparison | MUO | Comparison |  |  |  |  |
| IFN-γ | 15.2 | 10.5 | 146.6 | 15.1 |  | 0.003 | 1.29 | 0.1960 |
| IL-2 | 3.5 | 9.8 | 20.9 | 206.6 |  | -0.007 | -1.71 | 0.0880 |
| IL-6 | 10.7 | 17.2 | 9.4 | 82.8 |  | -0.013 | -1.74 | 0.0813 |
| IL-15 | 9.0 | 33.0 | 60.2 | 168.7 |  | -0.004 | -1.67 | 0.0943 |
| IL-10 | 8.5 | 8.5 | 0.0 | 49.6 |  | -0.037 | -1.88 | 0.0601 |
| TNF-α | 6.1 | 6.1 | 0.8 | 52.2 |  | -0.009 | -1.44 | 0.1505 |
| *** *p<0.001*, ** *p<0.01*, * *p<0.05* | | | | | | | | |

| Table S4: Discriminatory power of CSF cytokine concentrations in dogs with MUO and SRMA | | | | | | |
| --- | --- | --- | --- | --- | --- | --- |
| *This table provides the results of the CSF cytokine measurements of the two-sample Wilcoxon rank-sum (Mann-Whitney U) test evaluating for differences between meningoencephalitis of unknown origin (MUO) and steroid-responsive meningitis-arteritis (SRMA). For each cytokine (IFN-γ, IL-2, IL-6, IL-15, IL-10, and TNF-α), median concentrations (pg/mL) and interquartile ranges (IQR) are reported for both groups. The z-statistic represents the standardized test statistic of the Wilcoxon rank-sum test, with positive values indicating higher ranks in MUO and negative values indicating higher ranks in SRMA. The p-value denotes the two-sided probability of observing the test result under the null hypothesis of no difference between groups.* | | | | | | |
| CSF Cytokine | Median | | IQR | | z-statistic | *p*-value |
|  | MUO | SRMA | MUO | SRMA |  |  |
| IFN-γ | 110.9 | 14.6 | 73.4 | 40.0 | 4.823 | 0.0000 |
| IL-2 | 3.5 | 5.0 | 0.0 | 4.9 | -2.531 | 0.0114 |
| IL-6 | 3.7 | 460.6 | 34.4 | 1165.5 | -3.447 | 0.0006 |
| IL-15 | 9.0 | 9.0 | 0.5 | 4.5 | -1.001 | 0.3166 |
| IL-10 | 171.2 | 8.5 | 37.0 | 0.0 | 5.669 | 0.0000 |
| TNF-α | 6.1 | 6.2 | 0.0 | 2.3 | -1.678 | 0.0933 |

| Table S5: Discriminatory power of serum cytokine concentrations in dogs with MUO and SRMA | | | | | | |
| --- | --- | --- | --- | --- | --- | --- |
| *This table provides the results of the serum cytokine measurements of the two-sample Wilcoxon rank-sum (Mann-Whitney U) test evaluating for differences between meningoencephalitis of unknown origin (MUO) and steroid-responsive meningitis-arteritis (SRMA). For each cytokine (IFN-γ, IL-2, IL-6, IL-15, IL-10, and TNF-α), median concentrations (pg/mL) and interquartile ranges (IQR) are reported for both groups. The z-statistic represents the standardized test statistic of the Wilcoxon rank-sum test, with positive values indicating higher ranks in MUO and negative values indicating higher ranks in SRMA. The p-value denotes the two-sided probability of observing the test result under the null hypothesis of no difference between groups.* | | | | | | |
| Serum Cytokine | Median | | IQR | | z-statistic | *p*-value |
|  | MUO | SRMA | MUO | SRMA |  |  |
| IFN-γ | 15.2 | 10.5 | 146.6 | 41.7 | 1.190 | 0.2339 |
| IL-2 | 3.5 | 270.5 | 20.9 | 920.2 | -3.744 | 0.0002 |
| IL-6 | 10.7 | 139.8 | 9.4 | 419.5 | -4.895 | 0.0000 |
| IL-15 | 9.0 | 365.0 | 60.2 | 851.4 | -4.600 | 0.0000 |
| IL-10 | 8.5 | 8.5 | 0.0 | 35.5 | -2.525 | 0.0116 |
| TNF-α | 6.1 | 108.8 | 0.8 | 348.9 | -3.780 | 0.0002 |
